# A rapid method for assessing the accumulation of microplastics in the sea surface microlayer (SML) of estuarine systems

**DOI:** 10.1038/s41598-018-27612-w

**Published:** 2018-06-21

**Authors:** Zachary T. Anderson, Andrew B. Cundy, Ian W. Croudace, Phillip E. Warwick, Omar Celis-Hernandez, Jessica L. Stead

**Affiliations:** 1 Bermuda Institute of Ocean Sciences, Ferry Reach, St.George’s GE 01, St.George’s, Bermuda; 2 School of Ocean and Earth Science, University of Southampton, Southampton, SO14 3ZH UK

## Abstract

Microplastics are an increasingly important contaminant in the marine environment. Depending on their composition and degree of biofouling, many common microplastics are less dense than seawater and so tend to float at or near the ocean surface. As such, they may exhibit high concentrations in the sea surface microlayer (SML – the upper 1–1000 μm of the ocean) relative to deeper water. This paper examines the accumulation of microplastics, in particular microfibres, in the SML in two contrasting estuarine systems – the Hamble estuary and the Beaulieu estuary, southern U.K., via a novel and rapid SML-selective sampling method using a dipped glass plate. Microplastic concentrations (for identified fibres, of 0.05 to 4.5 mm length) were highest in the SML-selective samples (with a mean concentration of 43 ± 36 fibres/L), compared to <5 fibres/L for surface and sub-surface bulk water samples. Data collected show the usefulness of the dipped glass plate method as a rapid and inexpensive tool for sampling SML-associated microplastics in estuaries, and indicate that microplastics preferentially accumulate at the SML in estuarine conditions (providing a potential transfer mechanism for incorporation into upper intertidal sinks). Fibres are present (and readily sampled) in both developed and more pristine estuarine systems.

## Introduction

Microplastics (MPs), such as microbeads found in cosmetics and fibres from clothing or fishing activity, are an emerging and increasingly important anthropogenic contaminant in the marine environment^1^. Depending on their composition and degree of biofouling many common MPs are less dense than seawater so tend to float at or near the ocean surface^2^. This, coupled with the hydrophobicity of many common plastics, means that MPs may exhibit high concentrations in the sea surface microlayer (SML – defined conventionally as the upper 1–1000 μm of the ocean) relative to deeper water^3,4^. The SML is a habitat for a diverse array of marine organisms such as microscopic algae and bacteria, and microplastic accumulation in this layer may generate adverse physical and chemical (e.g. via leaching of adsorbed organic contaminants, or of plastic additives such as phthalates) impacts on these biota, and those organisms which feed on them^5–8^, with potential also for trophic transfer^6^.

Approximately 80% of marine plastics are derived from land-based anthropogenic sources (including beach litter)^9^, and estuaries have been recognised as major transfer pathways, and potentially sinks, for microplastics (e.g.^10^). Estuarine systems have a well-known filtering role for a range of anthropogenic contaminants via particle flocculation and other mechanisms, moderating the supply of these contaminants to the open ocean. Fine sediment-dominated intertidal and subtidal estuarine sedimentary systems (tidal and subtidal flats, saltmarshes, lagoons and mangroves) can act as important short-term and longer-term sinks for entrained and adsorbed contaminants (including MPs). Estuaries are also highly productive biological environments in terms of both primary productivity, and as nursery sites for fish and shell fisheries. As primary locations for urbanisation, shipping and industrial development, estuaries receive contaminant inputs (including MPs) from a range of local and upstream (and potentially downstream, i.e. marine) sources, including treated and untreated sewage, urban run-off, industry, fishing and shipping sources. While a number of studies have documented MP concentrations and distribution in estuarine and coastal systems (e.g^11–15^.) the accumulation of MPs in the SML in estuarine systems has only recently been considered^16^, and the potential impacts of SML accumulation on estuarine trapping and dispersion processes for MPs remains poorly constrained.

**Figure 1:**
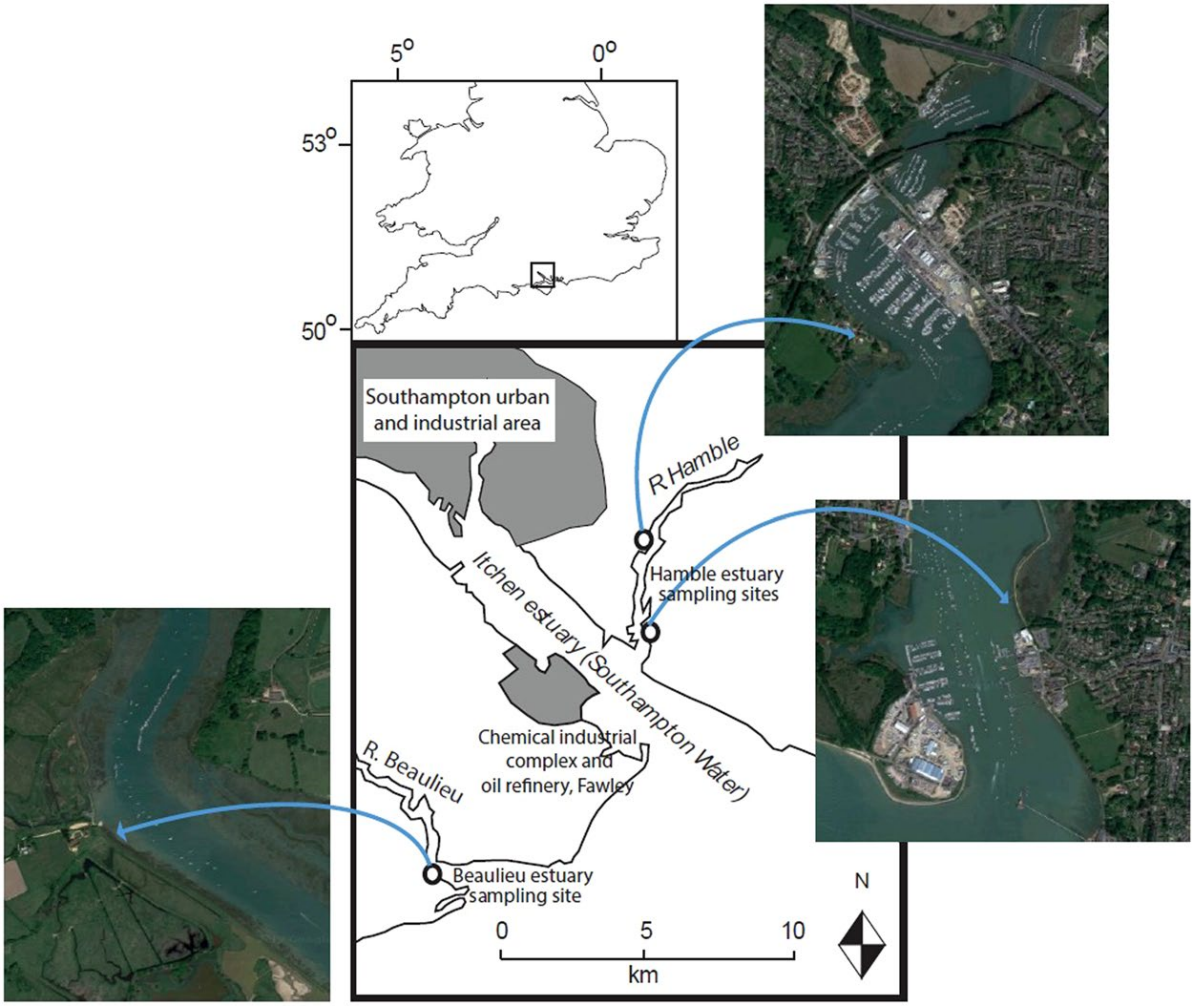
Study area: Beaulieu estuary and the Hamble estuary, southern UK. Aerial photographic images show detail of within-estuary sampling locations. Aerial photographic imagery Copyright 2017 Google. Map data Copyright 2017 Google.

Here we examine the accumulation of MPs in the SML from two contrasting estuarine systems – the Hamble estuary (part of the highly urbanised, developed and industrialised Southampton Water system), and the less developed Beaulieu estuary, both part of the Solent estuarine system in the southern U.K. (Fig. 1). A novel and rapid SML-selective sampling method for MPs, using a dipped glass plate, is proposed and evaluated in comparison with previously reported methods. Results are assessed in terms of estuarine transfer and trapping of MPs, and the importance of the SML as an accumulator of MPs.

## Results

A total of 84 MP fibres were identified in the study, from three sample sites over two sampling days (Table 1, Fig. 1). Three blank (control) filters revealed that significant contamination levels occurred during sample preparation and analysis; twelve fibres were found on each filter. However, virtually all were transparent (non-coloured) fibres, with just one small red fibre 0.17 mm in length identified. All transparent fibres found in environmental samples were therefore discounted for subsequent numerical analyses due to the strong possibility of their presence arising from sample contamination. The single red fibre in 1.5 litres of milli-Q water gives a hypothetical sampling error of 0.67 fibres per litre of sample, which was applied to calculate a lower bound for concentration values (Table 1). The dipped glass plate sampling method was found to sample to a water depth of between 114 and 190 μm, while sieve sampling collected SML samples 700–745 μm thick. Below, we examine the results in terms of: (a) the concentrations of MPs sampled using glass plate, sieve, surface skim and bulk water sampling methods; and (b) the characteristics of the MPs sampled.

**Table 1:** Identified MP fibres by method, showing number and colour of fibres, mean length and calculated fibre abundance.

Collection date	Site	Sample			Number of fibres				Mean length (mm)	Fibre abundance	
		Type	Vol. (mL)	Thickness (μm)	Blue	Red	Black	Tot.		(MPs/L)	(MPs/m^2^)
Day 1 21/06/17	1	Glass	314	—	11	4	1	16	0.83	*93*	*12*
		Sieve	315	—	6	2	0	8	1.35	—	—
		Skim	1270	—	6	0	0	6	0.86	4.7	—
		Bulk	2204	—	1	0	0	1	0.67	0.5	—
Day 2 29/06/17	1	Glass	190	152	5	2	1	8	0.96	42.1	6.4
		Sieve	499	745	1	1	1	3	0.75	6.0	4.5
		Skim	1135	—	0	2	2	4	0.81	3.5	—
		Bulk	2095	—	0	2	0	2	1.46	1.0	—
	2	Glass	114	91.2	1	1	1	3	0.73	26.3	2.4
		Sieve	469	700	0	1	1	2	1.03	4.3	3.0
		Skim	1096	—	3	2	1	6	0.99	5.5	—
		Bulk	1890	—	3	2	0	5	1.19	2.6	—
	**3**	Glass	190	152	2	0	0	2	0.29	10.5	1.6
		Sieve	492	734	2	1	2	5	1.17	10.2	7.5
		Skim	1135	—	2	0	1	3	2.01	2.6	—
		Bulk	2058	—	9	1	0	10	1.38	4.9	—
TOTALS					**52**	**21**	**11**	**84**			

Sites 1 and 2 = Hamble, site 3 = Beaulieu. For site 1, Day 1, fibre abundance values (italicised) for the glass plate sampling method were calculated using a modified equation to allow for dilution of samples by wash water (see Sampling and Analytical Methods). This dilution step was not performed on Day 2.

### Environmental concentrations, and sampling method comparison

**Figure 2:**
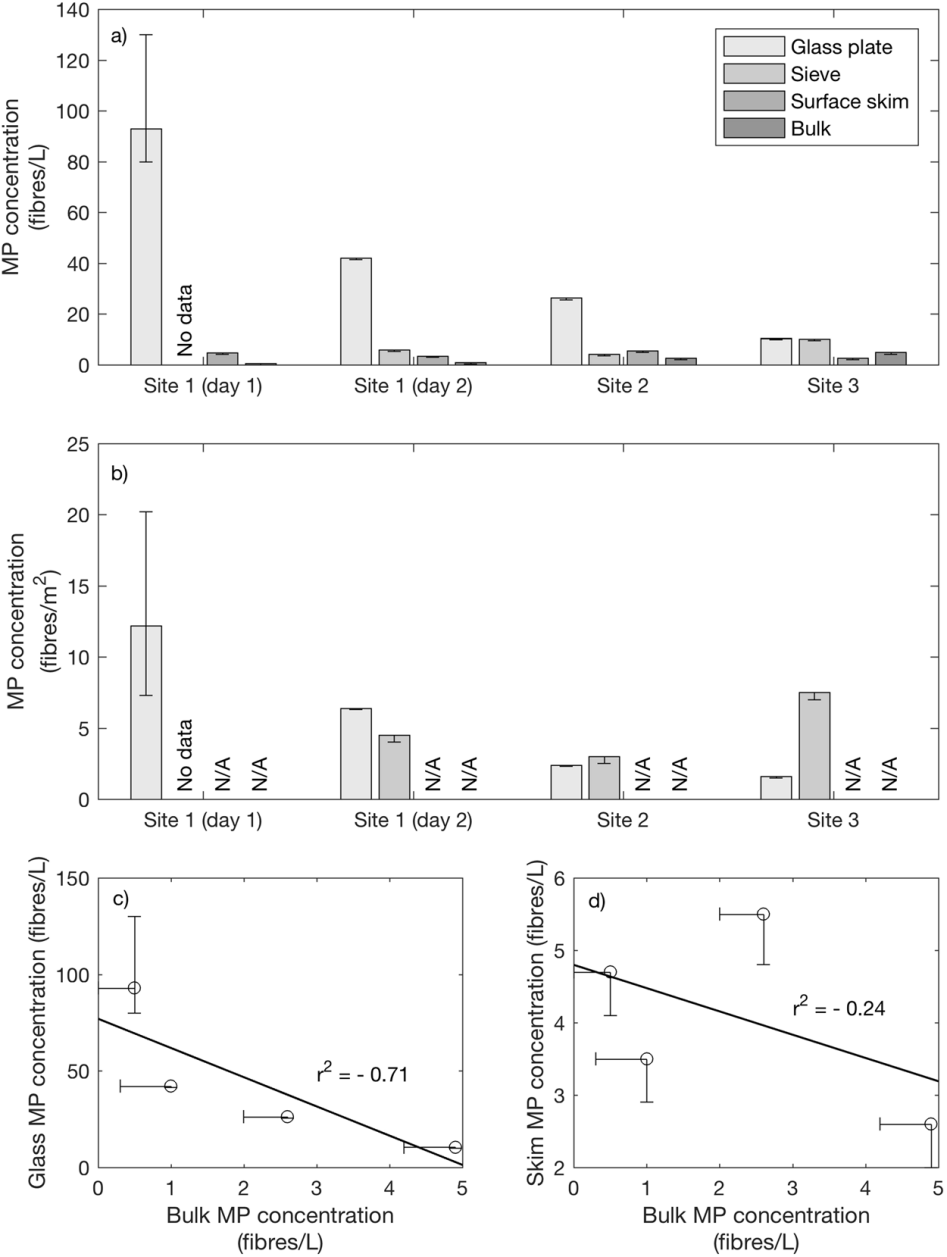
Microplastic fibre concentrations, calculated volumetrically for all samples **(a)** and spatially for SML samples **(b)**, show clear discrepancy between methods. Glass plate sample MP concentrations are highest at all locations, with bulk MP concentrations lowest for all but Site 3. A linear least squares regression comparing glass plate to bulk MP concentrations reveals a negative correlation **(c)**. A similarly negative but far weaker correlation exists between surface skim and bulk MP concentrations **(d)**. N = 4.

Dipped glass plate sample MP concentrations were higher than from all other sampling methods (sieve, surface skim and bulk water sample collection) at all sites (Fig. 2), apart from the Beaulieu site (where concentrations from the glass plate sampling method were similar, in MPs/L, to those determined via sieve sampling), with a mean concentration of 43 ± 36 fibres/L. Sieve and surface skim samples had mean MP concentrations of 7 ± 3 fibres/L and 4 ± 1 fibres/L, respectively. Bulk sample MP concentrations were the lowest of all methods except at site 3, with a mean value of 2 ± 2 fibres/L. A Mann-Whitney U test (nonparametric) showed that the median glass plate sample MP concentration was greater than the median bulk water sample MP concentration, significant at the 5% confidence level (p = 0.03). A similar test comparing median values of glass plate and sieve samples from sampling day 2 also showed a difference significant at the 10% confidence level (p = 0.10). Mean spatial SML MP concentrations were calculated as 6 ± 5 fibres/m^2^ and 5 ± 2 fibres/m^2^ using the glass plate and sieve sampling methods, respectively. When statistically compared, there was no significant difference between the median values of the two methods (p = 0.40).

The concentration of MPs identified in glass plate samples from day 1 (93 fibres/L) is significantly greater than that from day 2 (42.1 fibres/L), even when considering the large errors in the day 1 calculations (Table 1). However, due to limited data, statistical analysis of temporal changes is not possible. A 25% decrease was observed in MP concentration for surface skim samples between day 1 and day 2, while bulk sample MP concentration doubled from 0.5 fibres/L to 1.0 fibres/L.

Considering only day 2 data, the concentrations of MPs in the glass plate and surface skim samples from the Beaulieu Estuary (10.5 and 2.6 fibres/L, respectively) are lower than from either of the Hamble Estuary sites. Conversely, the Beaulieu sieve and bulk sample MP concentrations (10.2 and 4.9 fibres/L, respectively) are 70–90% higher than the highest concentrations recorded at the Hamble sites. Microplastic fibre concentration from the sieve sample was ~5 times higher than that from the glass plate sample at Beaulieu, which is a much higher discrepancy than observed for the Hamble estuary SML samples.

### MPs characteristics

**Figure 3:**
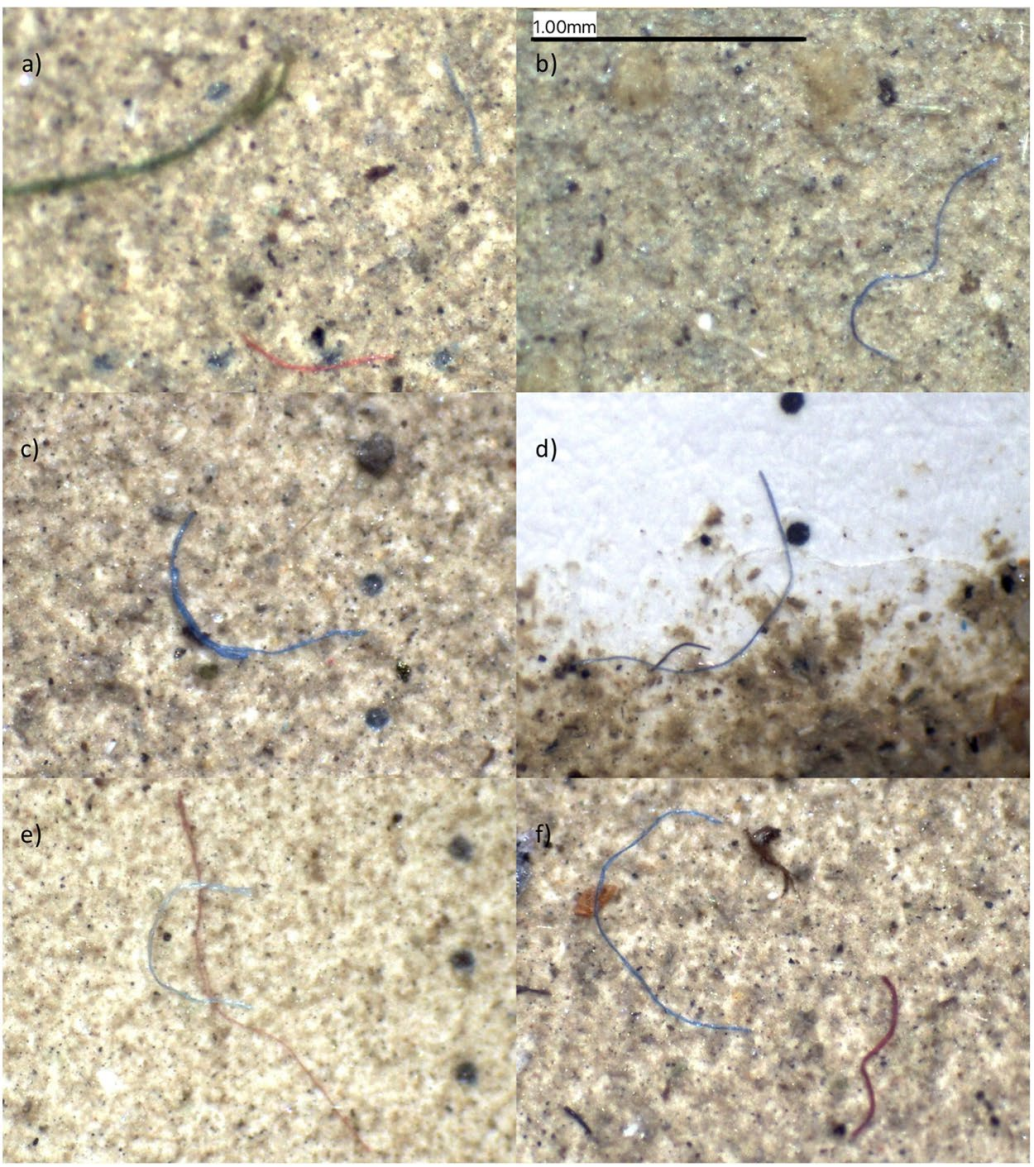
Examples of MP fibres identified using standard light microscopy at 35x magnification.

Standard light microscopy revealed that fibres were predominantly blue in colour (52), with 21 red and 11 black fibres also counted (Fig. 3). Fibres were generally smooth, with some examples exhibiting frayed ends or tapering. Multiple fibres were often found close together (e.g. Fig. 3f) or overlapping (e.g. Fig. 3c–e). The two blue fibres in Fig. 3c are clearly of the same origin and have retained coherence since their release into the environment, whereas others may have been agglomerated during sampling and filtration.

**Figure 4:**
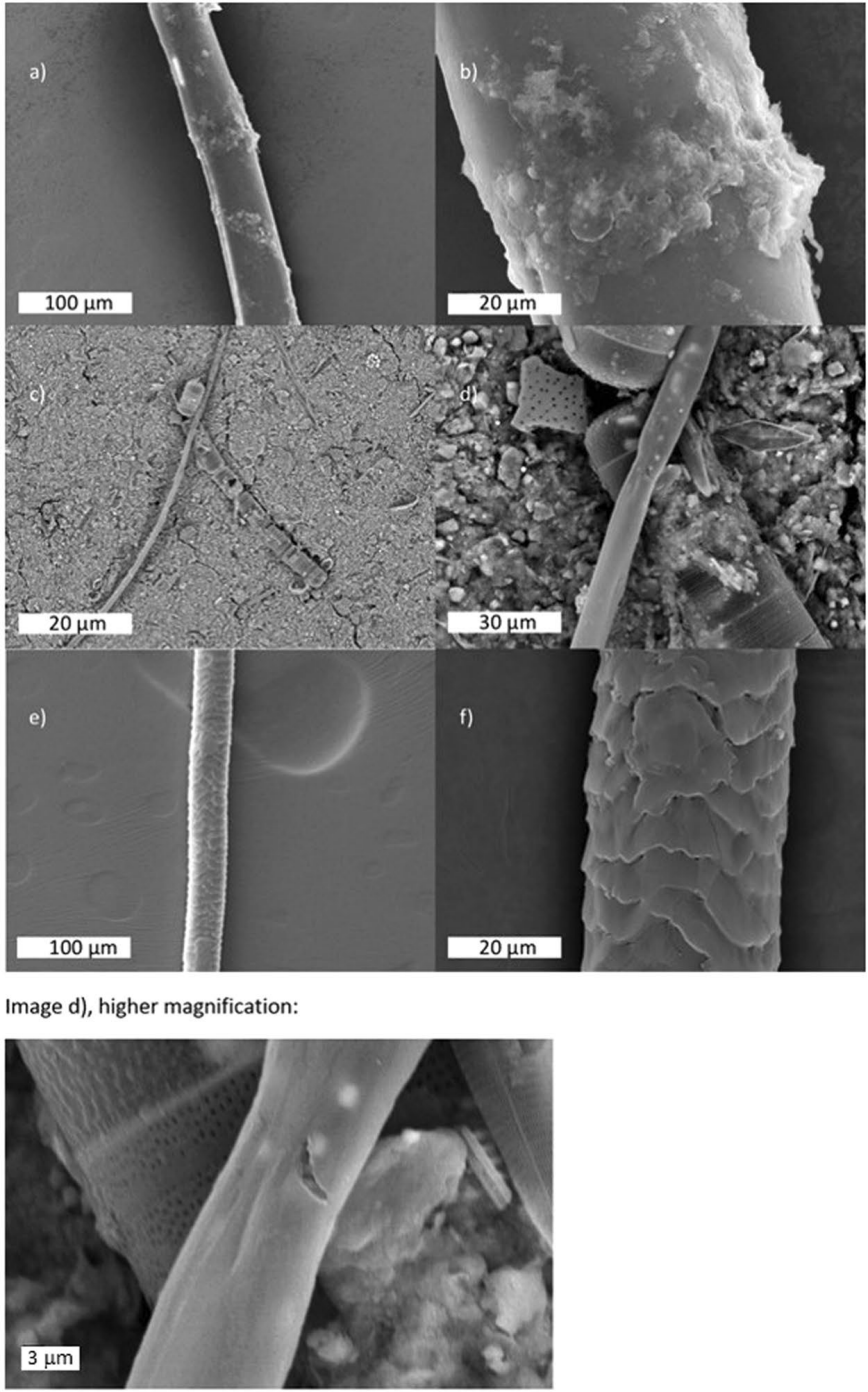
Scanning electron micrographs of (**a-b**) Plastic microfibre with adhered sediment, (**c–d**) Microfibre potentially associated with diatom chain, (**e–f**) Biological (scaled) fibre. Lower image shows higher magnification micrograph of fibre present in sample d).

Microplastic fibres were observed via SEM to have a generally smooth and non-weathered morphology. Sediment adhesion, largely of clay minerals, was clearly identified on fibres (Fig. 4a,b). The terminal diatom of a diatom chain appeared to be adhered to the surface of one MP fibre (Fig. 4c,d). Some possible weathering of this fibre is visible as microcracks and fracture structures close to the site of diatom adhesion. Cellular structures and regular rough surface structures from biological fibres were easily distinguishable under the SEM, such as the scaled texture observed in Fig. 4e,f.

### Fibre lengths

**Figure 5:**
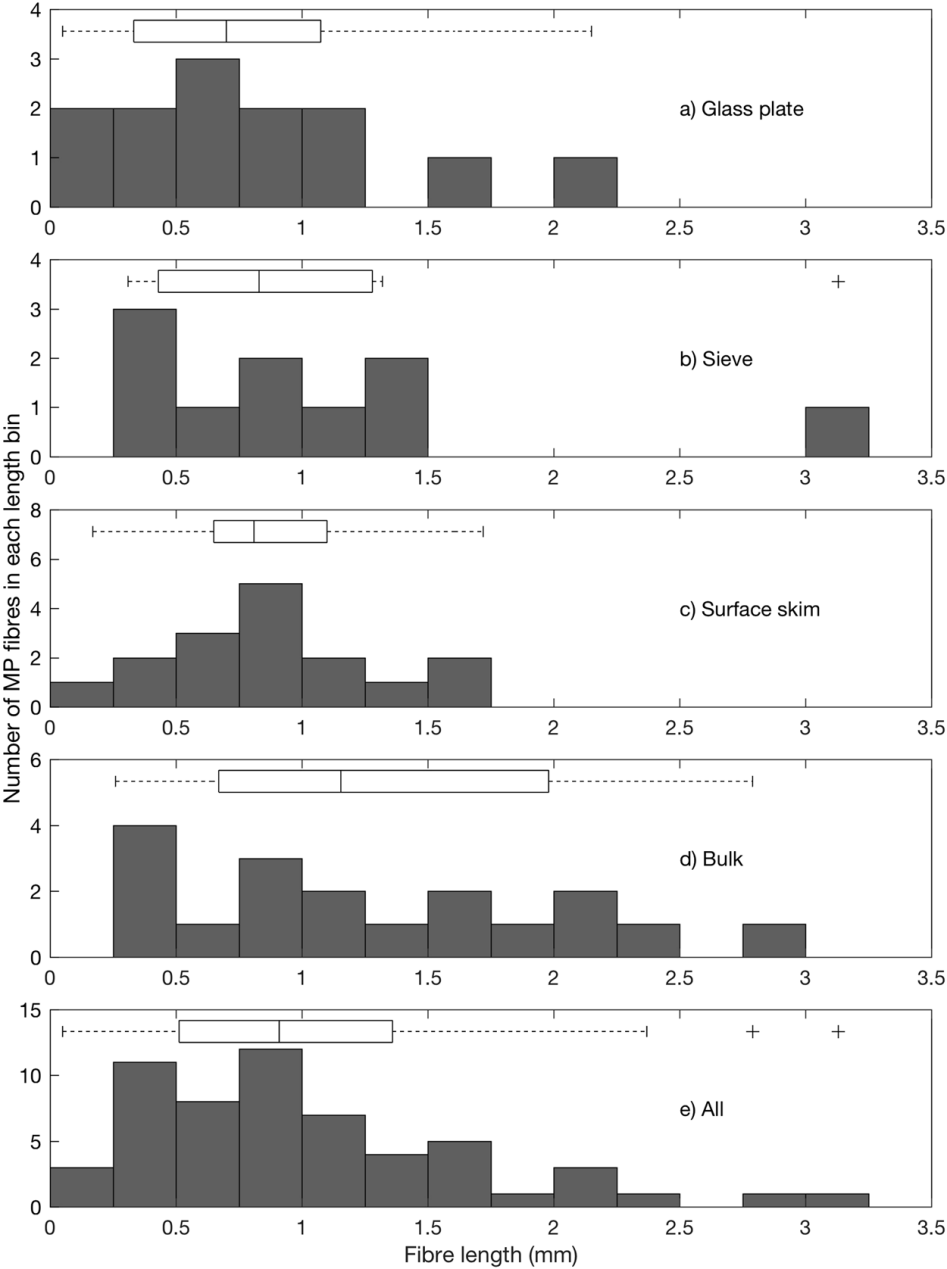
Comparison of fibre length as a function of sampling method reveals median fibre length and interquartile range are smaller for surface sampling methods (**a–c**) than bulk samples **(d)**. Glass plate **(a)** and sieve **(b)** data are from day 2 when no sample dilution occurred. Surface skim **(c)** and bulk **(d)** data are combined from both days. Data combined from a-d show fibre length distribution from all methods **(e)**. The central box plot line is the median, edges indicate the interquartile range, and whiskers extend to the data points not considered outliers (+).

The lengths of fibres identified range from 0.05 to 4.5 mm, all below the broadly used upper size limit for classification as MP (5 mm), with a mean of 1.05 ± 0.76 mm and modal length between 0.25 and 1 mm, depending on sampling method. Smaller fibres were not counted due to the distinct likelihood of incorrect identification. Fibres collected by glass plate and sieve on day 1 were excluded from further statistical analyses due to sample dilution with surface skim water. Fibre length does not follow a normal distribution, either when separated by sample type or when analysed together (Kolmogorov-Smirnov test, 6 × 10^−16^ ≤ p ≤ 8.5 × 10^−4^, depending on sample type). All distributions display a positive skew (Fig. 5). The sieve sample is most skewed (1.78), largely due to a 3.13 mm long outlier, whereas the bulk sample displays least skew (0.34). A nonparametric statistical test shows that the median fibre length from glass plate samples is shorter than for bulk samples, significant to the 10% significance level (Mann-Whitney U test, p = 0.08). This discrepancy is less clear when comparing sieve to bulk samples (p = 0.24), and surface skim to bulk samples (p = 0.12). There is no statistically significant difference in median fibre length between surface samples; comparing glass plate to sieve, glass plate to surface skim, and sieve to surface skim yield p = 0.54, p = 0.47 and p = 0.90, respectively.

Ignoring outliers, bulk water MP fibre length has the highest median (1.15 mm), range (2.53 mm), interquartile range (1.31 mm), and 25th and 75th percentiles (0.67 mm and 1.98 mm, respectively) of all sampling methods used. Comparing the combined data from glass plate, sieve and surface skim samples with bulk water sample data suggests fibre length probability distribution differs between surface and bulk sampling methods, significant to the 10% significance level (two-sample Kolmogorov-Smirnov test, p = 0.10). Less significant values were revealed when comparing glass plate to bulk (p = 0.23), sieve to bulk (p = 0.35) and surface skim to bulk (p = 0.16). Conversely, intercomparison of surface sampling methods displayed no statistically significant difference at all (0.58 ≤ p ≤ 0.91, depending on the methods compared). Temporal and spatial changes in fibre length with different methods were not assessed, as data were too limited for meaningful statistical analysis.

## Discussion

A number of authors have previously noted the important role played by the SML in contaminant cycling^17^. High microbial activity produces a “sticky” microgel characteristic of the SML^18^, which also contains extremely elevated concentrations of hydrophobic contaminants such as POPs, thereby providing an ideal environment for contaminant adsorption to MP particles^19^, and transfer to SML-dwelling organisms. In this study, significantly higher MP concentrations were found for glass plate collected samples than for bulk samples, which supports the hypothesis that MPs preferentially accumulate at the SML in estuarine conditions. This agrees with other studies from coastal and open ocean environments that have found higher MP concentrations at the SML than at depth^3,4,20,21^. Mean SML MP fibre concentrations found in this study using the sieve collection method (7 ± 3 fibres/L) are comparable to those of coastal waters given in other studies using the same method: 11 ± 8.2 fibres/L and 4.5 ± 4.1 fibres/L were reported by Song *et al*. in their 2015^20^ and 2014^4^ studies, respectively, although Gray *et al*. report significantly higher values of 30.8 ± 12.1 particles/L in Winyah Bay, South Carolina^16^.

In areas where wave motion and strong turbulent mixing is limited, MPs will likely accumulate within the SML^22^. During calm water conditions in partly-enclosed estuaries, the flooding tide will traverse open mudflat environments, potentially remobilising and incorporating surface deposited MPs into the SML and transporting these into upper tidal flat and saltmarsh environments. This offers a potential transfer mechanism into upper intertidal mudflat and saltmarsh sinks, which may be enhanced in systems such as Southampton Water where frictional effects and a distorted tidal curve cause an extended slack water period, and drive lateral suspended sediment transport from the main channel to the intertidal zone^23^. These sinks, particularly saltmarshes where vegetation may have a direct interceptive or “baffle” effect for MP deposition, may act as important short and medium term MP accumulation sites, although MP trapping processes in estuarine flats and saltmarshes are not currently well-constrained.

Only data for MP fibres are presented here, which is at least partly a methodological effect (the high sediment load on the filters preventing strong positive identification of beads and non-fibrous particles or MP fragments against the more common lithic and biological fragments). However, previously published data on MP in the Hamble estuary (collected by plankton net trawl) in 2013 showed that >80% of particles collected were fibres^15^. While spatial (or indeed temporal for site 1) differences in MP concentrations cannot be assessed in detail using the data (and the number of within-estuary replicates) provided, it is notable that both Beaulieu (less-developed) and Hamble (part of a wider urbanised/industrialised system, with significant local recreational boating and marina activity) sampling areas showed MP presence. The Beaulieu site recorded the highest bulk and sieve sample MP concentrations (4.9 fibres/L and 10.2 fibres/L, respectively), with bulk concentration even exceeding that of the surface skim sample (2.6 fibres/L). This highlights the ubiquity of MPs even in relatively pristine environments (the Beaulieu river does receive some sewage inputs, from three WWTWs which discharge wastewater into the Beaulieu and its tributaries, although these input an order of magnitude lower dry weather discharge than those in the Hamble). The rainfall in the sampling area prior to sampling on day 2 (Table 2) may have enhanced run-off and sewage contributions to the Beaulieu site. The different aspects of the sites in relation to prevailing wind direction may also enhance MP accumulation at the more sheltered Beaulieu site, which will experience less fetch than the downstream Hamble site (site 2) in particular. However, volumetric MP abundances derived using each sampling method at the Beaulieu site are more similar than those at the Hamble sites, which may in fact indicate increased local water column mixing here. Observations on fibre morphology suggested that the estuarine residence times of some fibres were long enough for sediment adhesion and possibly biofouling to occur.

**Table 2:** Meteorological conditions on sampling days recorded at Southampton Weather Station, located at 50° 53.98 N, 1° 23.73 W (source: UK Meteorological Office).

Date	Weather	Mean air temperature (°C)	Mean wind speed (km/h)	Mean wind direction (°)	Rainfall in past 72 hours (mm)
21/06/17	Sun	23.9	7.4	137 (SE)	0
29/06/17	Cloud and light rain	14.2	3.1	284 (WNW)	12.8

The glass plate sampling method enabled sampling of the upper *ca*. 100 μm of the estuarine water surface, the thinnest SML sample of all surface sampling methods, and gave the highest MP concentrations volumetrically. Whilst this method collects a smaller sample volume per dip than with a sieve, it samples a far thinner surface layer: roughly 100–200 μm using the sampling procedure outlined in this study, compared to 700–750 μm with the sieve method. As the SML is typically ~100 μm thick^21^, the glass plate sampling method has the distinct advantage of producing a sample that will be far more representative of the SML, without interference or dilution from deeper layers. It is also inexpensive, and easy to operate. It does require however relatively sheltered (still water) conditions, as strong wind-wave action may act to mix and distribute the MPs through the water column (and temporarily disrupt the SML). Sample collection may also be problematic from a floating platform or boat during moderate to high sea states as it may be difficult to control the rate at which the plate is pulled through the surface^24^. The SML sampling methods used here all facilitate easy collection of MPs without use of heavy or specialist equipment. Compared with sieve samples and the more common net-tow samples, those collected by glass plate contain relatively small amounts of sediment and biogenic material, which tend to hinder MP identification. Additionally, SML MP abundance may be calculated as a volumetric or spatial concentration. However, Song *et al*.^4^ reported underrepresentation of larger sediment particles (1–5 mm diameter) with SML sampling techniques, which may also be true for fibres. Median fibre length from glass plate samples was observed here to be shorter than in bulk water samples, significant to the 10% significance level. Notably, for the size data presented here, over 10% of fibres identified were shorter than 333 μm, which is the most commonly used net trawl mesh diameter^25^. This increases to over 17% when considering only SML fibres. Microplastic abundances given by surface trawl methods are therefore almost certainly underestimated, due to smaller particles passing through the mesh.

Despite precautions being taken to limit sample contamination effects during MP separation and analysis, transparent (non-coloured) fibres had to be excluded from the analysis due to their potential to be incorporated as contaminants during filtration, separation and analysis. Fibre concentrations presented are likely to therefore be conservative, additionally as smaller particles less than *ca*. 50 μm were unlikely to be resolved at the optical magnifications used to identify MPs. A number of previous studies have not recorded the implementation of precautionary measures to limit background fibre contamination. The control data recorded here, given the ubiquity of plastic fibres in particular in ventilation systems, indicate a need to quantify background contamination in all studies and to operate cautious sample handling in clean laboratory facilities when analysing environmental media which are likely to contain very low MP concentrations.

Previous experimental studies have shown microplankton ingestion of MP fibres^6^, beads^5^, and nanoplastic (<1 μm) particles^7^, and the current study indicates that further studies are required on the biological impacts of MPs accumulated in the surface microlayer of estuarine systems. Many laboratory studies of MP ingestion by organisms use concentrations that are far from being environmentally relevant – often millions of particles per litre – many orders of magnitude higher than found in this, and comparable, studies^5,26^. Experiments should therefore adjust their MP concentrations to derive more representative interpretations of the likely impacts on organisms.

### Sampling and analytical methods

The Hamble and Beaulieu estuaries were selected for study as they show broadly similar size and mixing characteristics, but contrasting degrees of development. Both estuaries are partially-mixed systems with flushing times of a few days. The Hamble estuary is a more developed estuary with significant local recreational boating and marina activity, is a designated eutrophic system, and is part of the urbanised and industrialised wider Southampton Water estuarine system. The Beaulieu estuary in contrast lies in the New Forest National Park, an area of predominantly open heath and bog with little agriculture and very few urban areas. Sampling sites within each estuary were chosen primarily for their sheltered locations and ease of shoreline access. All sites are significantly below the main estuarine mixing zone in each estuarine system, and show near fully marine salinities. Site 1, located 3.5 km upstream from the mouth of the Hamble river on the western bank directly opposite a large marina, was sampled on both sampling days (Table 3), with the first day primarily used to test and refine the glass plate sampling method, but also to give preliminary data on temporal variability in MPs concentrations and characteristics. Sampling was performed immediately in front of the slipway access to the waterfront area. Sampling site 2, two kilometres downstream from site 1 on the Hamble, was located 30 metres north of the Hamble-Warsash Ferry access boardwalk on the eastern side. The third site was located on the Beaulieu river, directly off the Southampton Yacht Club slipway on the outside western bank of a meander, two kilometres upstream from the main Solent channel. Sites 2 and 3 were accessed only on the second sampling day. Sampling was performed on 21/06/17 and 29/06/07 at the incoming face of the flooding tide in shallow water (<0.4 m depth) less than two metres from the shoreline, where the accumulation effect of resuspending MPs from the low tide line is hypothetically greatest. Samples were collected during calm conditions, in periods when boat traffic was absent, to ensure that the SML was relatively undisturbed.

**Table 3:** Sampling locations and times (in relation to low and high water), 21/06/17 and 29/06/17.

Date	Site	Description	Location	Sampling time	Low tide		First high tide	
					Time	Height (m)	Time	Height (m)
Day 1 21/06/17	1	Upstream Hamble	50° 52.845 N 1° 18.138 W	1530	1401	1.33	2031	4.53
Day 2 29/06/17	1	Upstream Hamble	50° 52.845 N 1° 18.138 W	1030	0811	1.02	1450	4.35
	2	Downstream Hamble	50° 51.337 N 1° 18.457 W	1130	0805	1.07	1448	4.29
	3	Beaulieu	50° 47.039 N 1° 24.753 W	1250	0807	0.70	1434	3.57

Four different sampling techniques were used to determine variations in MP concentrations within the upper 5 cm of surface water at potential shoreline MP accumulation zones (in areas protected from direct wave action). The SML was sampled using a stainless‐steel sieve and a glass plate, while surface skim and bulk water samples were also taken.

**Figure 6:**
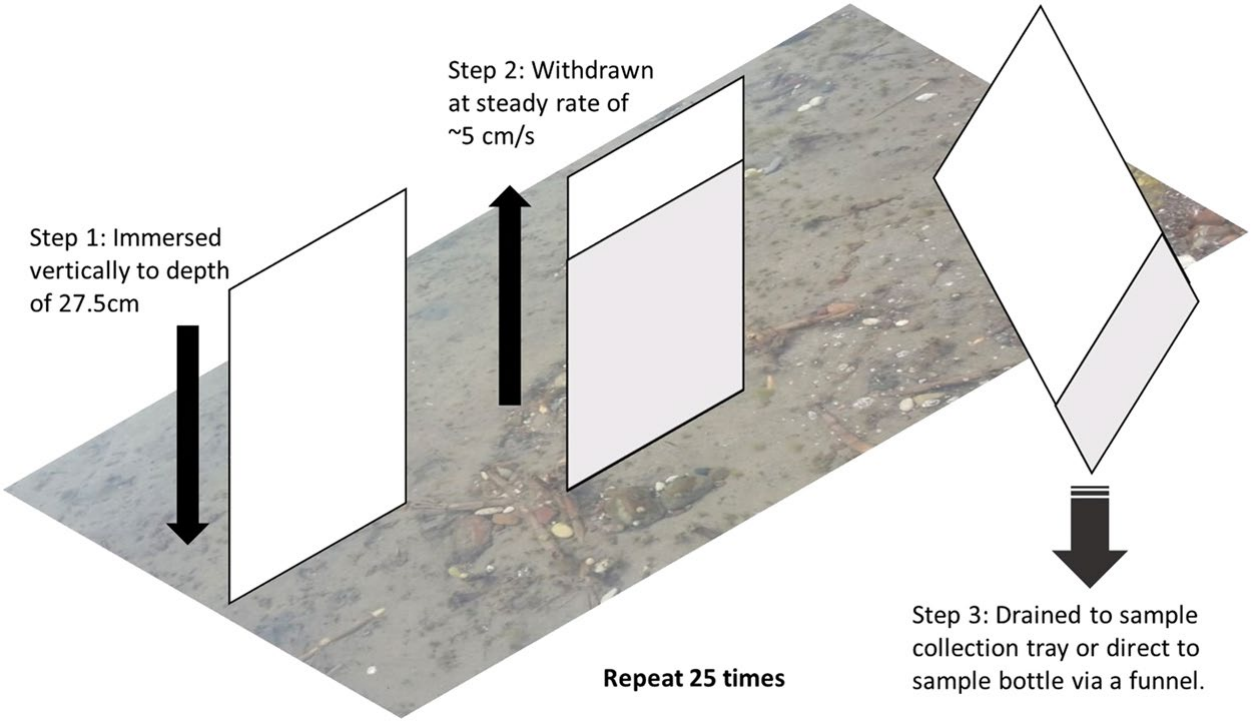
Schematic illustration of the glass plate sampling method used in the present study.

Glass plate sampling is commonly used to sample the SML^27,28^, though it is entirely novel as a method of investigating MP contamination. The method detailed at length by Cunliffe and Wurl^24^ was followed, with some additional amendments and improvements to the procedure. A 0.4 cm-thick glass plate, measuring 18.2 cm wide by 30 cm high, was immersed perpendicular to the water three times to remove potential contaminant MPs from the surface of the glass. The plate was immersed to a predetermined depth of 27.5 cm (Fig. 6), retaining 2.5 cm of handling space above the surface, to allow for reproducibility and precise calculation of the total surface area sampled. The plate was then withdrawn at a steady rate of ~5 cm/s and the adhered water sample immediately drained from both sides of the glass into a sample collection tray. This procedure was repeated for a total of 25 glass plate dips, over a water area of approximately 25 m^2^, collecting the subsequent sample water as a composite sample within the same collection tray. The tray was then emptied through a plastic funnel into a 500 mL polyethylene terephthalate (PET) storage bottle. The collection tray was then also rinsed with a small amount of local sea surface water, to make sure no MPs remained, and the rinse water added to the sample bottle. The tray, funnel and sample bottle were all pre-rinsed three times with surface water prior to sampling, for this and all subsequent methods. During day 2 sampling, samples were drained directly into the funnel and storage bottle from the glass plate.

Sea surface microlayer sampling was also undertaken using a Christison stainless-steel 1.18 mm mesh aperture sieve with an internal diameter of 18.5 cm. As with the glass plate SML sampling method, the method used for the sieve was similar to that from Cunliffe and Wurl^24^. The sieve was rinsed with surface water three times then gently touched to the surface of the water and slowly withdrawn, keeping it parallel to the water surface at all times. This differs from Cunliffe and Wurl^24^, who suggest immersing the sieve completely before slowly bringing it through the SML – a necessary procedure for shipboard operations, but which disturbs the ocean surface. The sieve was allowed to drain into a sample collection tray by tipping slightly immediately following withdrawal. Any remaining water was shaken off into the tray after ten seconds. This process, as before, was repeated 25 times. The sample was then drained from the tray into a 500 mL PET sample bottle and the tray rinsed with surface water, which was subsequently collected. Use of a 1.18 mm mesh sieve in initial field sampling was observed to inhibit complete dispensing of the SML sample, due to high surface tension on the adhered sample between the mesh. A larger sieve aperture was therefore deemed necessary, and following brief laboratory testing a 2 mm-aperture sieve was used during the day 2 sampling. As for the glass plate sampling method, day 2 samples were drained directly (via a plastic funnel) into the PET sample bottle.

Additional samples were taken on both sampling days directly into 2 L PET sample bottles. Surface water was collected by gently skimming the upper ~5 mm of the water surface through the mouth of the sample bottle until 1–2 L of water was collected. Bulk water samples were also taken, excluding surface water; a closed bottle was submerged and the opening brought to ~5 cm depth before removing the lid and filling with ~2 L of bulk water. The lid was replaced while still submerged before bringing the bottle to the surface. All water samples were kept refrigerated at 3–5 °C following collection until use.

Water samples were vacuum-filtered through 47 mm-diameter Thermo Scientific 0.45 μm Nalgene™ membrane filters, in a chemical preparation laboratory at the National Oceanography Centre (Southampton). All glassware was rinsed with water and rinsed in triplicate with milli-Q water prior to use and between samples, though not between filters of the same sample. Due to heavy biological and sediment loads and large sample volumes, multiple filters were needed for all but the glass dipping method samples. Aliquots of 250–500 mL of sample, depending on water clarity, were measured using a 500 mL glass measuring cylinder and transferred to the glass filter cup. A dampened larger diameter 0.45 μm filter was placed over the filter cup during filtration to limit contamination from dust, which may contain MP fibres. After filtration the filter was removed with clean forceps and stored in a covered plastic petri dish. For the final aliquot of each sample, the cylinder and filter cup were both rinsed with milli-Q water, which was then passed through the same filter. Blanks were prepared in triplicate by filtering 500 mL of milli-Q water in the same way as for samples. These were then analysed to provide an estimate of contamination through sample preparation and handling.

Filters were analysed under 35x magnification using a Leica EZ4 binocular dissecting microscope. Filters remained covered except during analysis to avoid prolonged exposure to atmospheric contamination from dust. A clean workspace was maintained by keeping all surfaces and equipment such as probe and forceps clean with lint-free wipes. Cotton clothing was worn as an additional precaution against contamination from MP fibres. The gridded filters were examined methodically from left to right along the first row, right to left along the second row and so on, to prevent double-counting of MPs. Microplastic fibres were counted and categorised according to their colour and length, as in numerous other studies (e.g.^25^). Other types of MP debris (e.g. fragments, particles etc.) were not identified or counted, due to high sediment load on the filters preventing strong positive identification against the more common lithic and biological fragments. Detritus on the filters was carefully moved to ensure fibres were not missed. All fibres were digitally imaged for later reference and accurate determination of fibre length. Some larger fibres were collected using forceps and stored in low-density polyethylene ziplock bags for later analysis by scanning electron microscopy (SEM).

Fibrous materials were determined as MP by careful identification of exclusive characteristics: shape, texture, colour and other physical properties. Larger, thicker MP fibres were identified by their clear cylindrical shape and uniform thickness; a smooth surface was also used as an identification tool. Frayed fibre ends were a clear but accepted deviation from this that was used to help identify MP fibres. Vibrantly coloured fibres (i.e. red or blue) were also relatively easy to identify as MP, whereas classification of black or clear fibres depended on other features. Fibres non-homogenous in colour were treated with equal suspicion. Any fibre that was in a notably raised position above the filter surface, or that appeared to sit on top of background sediment as opposed to with it, was considered as potential contamination by air-borne fibres and was not counted or recorded. Tweezers and probes were used to manipulate fibres; if gentle bending or prodding caused a fibre to break then it was not counted as plastic. Conversely, elasticity displayed by springing or bouncing when touched suggested plastic composition.

Observations of well-defined characteristics of biological material were used to rule out as MP certain fibres. Such biological features included clear cellular structure or segmentation along the length of a fibre, brittleness, and tapered shape. Care was taken to ensure cellular structures observed did not belong to adhered biofouling organisms and biofilms. Man‐made natural fibres such as cotton, wool and flax possess different physical qualities to those of plastic fibres, which can be used to identify them as non-plastic; none are perfectly cylindrical and some have visible cellular structure. Only fibres determined with certainty to be MPs were counted, to obtain a conservative but confident estimate of MP concentration.

A Leo 1450VP SEM was used to validate the identity of previously collected fibres as either MP fibres or biological in nature, as well as examine fibre surface morphology for signs of degradation, sediment adhesion or biofouling. The SEM was operated under 500x, 1000x and 5000x magnification. Variable pressure SEM mode was used when electrical charging effects on the sample presented imaging issues for certain fibres.

The SML thickness h (μm) sampled using each technique was calculated as follows^24^:

1 $$h=\,\frac{{10}^{4}\,.\,V}{N.A}$$

where V is sample volume (mL), N is the number of glass plate or sieve dips per sample, and A is the surface area sampled per dip (cm^2^). This is the total immersed area of both sides of the glass plate, or the internal mesh area of the sieve.

Site 1 SML samples collected via sieve and glass could not be directly compared between sampling days, owing to methodological changes, in particular the use of a different mesh-aperture sieve and dilution of the samples with surface skim rinse water. To enable comparison, an effective (undiluted) SML MP concentration for the glass plate method sample from day one was calculated using the observed glass sample concentration, the SML sample thickness, and the surface skim rinse water concentration. This required two assumptions. First, that the thickness of the SML sample collected on day one via the glass plate method was similar to that collected on day two. This thickness was calculated as the mean value of the three other samples. Secondly, that the concentration of MPs in the rinse water was equal to that collected at the same location using the surface skim bottle collection method. The glass plate method SML MP volumetric concentration *C*_*V*_ (fibres/L) for day one was calculated using the formula:

2 $${C}_{v}\,=\,\frac{1000n-{C}_{ss}(V-{\bar{V}}_{g})}{{\bar{V}}_{g}}$$

where *n* is the number of identified MP fibres in the sample, *C*_*ss*_ (fibres/L) is the day 1 surface skim sample concentration, *V* is the sample volume (mL), and ${\bar{V}}_{g}$ (mL) is the mean glass plate volume collected on day 2. Spatial volumetric concentration *C*_*s*_ (fibres/m^2^) for the day 1 glass plate sample was calculated using:

3 $${C}_{s}=\frac{10{C}_{v}.{\bar{V}}_{g}}{N.A}$$

Errors in these calculations were considerably greater than for those from day 2, due to the additional propagation of uncertainty in ${\bar{V}}_{g}$. Lower and upper error bounds were taken as the minimum and maximum glass plate sample volumes collected on day 2, respectively.

A Mann-Whitney U test was used to determine statistically significant differences between average (median) concentrations for different methods, assuming a non-normal distribution due to the low number of samples. Linear least-squares regressions were used to determine any possible positive or negative correlations between MP concentrations obtained using different sampling methods. Fibre lengths were tested for normal distribution with a Kolmogorov-Smirnov test, with subsequent analysis using Mann-Whitney U tests to determine if statistically significant differences in median fibre length between sampling methods existed. A two-sample Kolmogorov-Smirnov test was used to compare fibre length probability distributions from different surface and bulk sampling methods. Mean values are presented with an error given as one standard deviation.
